# Concentrated Growth Factor: A Novel Platelet Concentrate for Revascularization of Immature Permanent Teeth—A Report of Two Cases

**DOI:** 10.1155/2020/1329145

**Published:** 2020-08-15

**Authors:** M. S. Nivedhitha, Benoy Jacob, Aishwarya Ranganath

**Affiliations:** Department of Conservative Dentistry and Endodontics, Saveetha Institute of Medical and Technical Sciences, No. 162, Poonamalle High Road, Chennai, 600077 Tamil Nadu, India

## Abstract

This article elucidates the utilization of a novel platelet concentrate-concentrated growth factor (CGF) for rapid and successful healing outcome in regenerative endodontics. This case report focusses on two cases: 23-year-old and 21-year-old patients with incomplete root formation and periapical lesion. Case 1 and case 2 are classified as stage IV and stage II, respectively, in accordance with Cvek's classification of open apex and had varied outcomes. The extent of open apex, root dentin thickness, and lesion were assessed using periapical radiograph and CBCT. Revascularization procedure was carried out after obtaining patient consent. Following bleeding induction, CGF was prepared, placed, and condensed using pluggers in the root canal space, followed by the placement of mineral trioxide aggregate (MTA) up to the level of CEJ. At 1-year follow-up, apical closure with increased root dentin thickness and reduced periapical radiolucency was evident.

## 1. Introduction

The developing dentition is perpetually subjected to various mechanical or biological insults pertaining to trauma, caries, and diverse developmental anomalies [1]. There has been tremendous research aimed at the management of nonvital immature anterior permanent teeth to reestablish the pulp-dentin complex. Since the nonvital tooth is devoid of blood supply, the root fails to develop and is subjected to fracture in the long run. Cvek in 1992 classified five stages of root development according to the width of the apical foramen and length of the root where he described teeth with wide divergent apical opening estimated to be less than half, half, two-thirds, nearly completed, and finally teeth with closed apical foramen and completed root development [2].

Revascularization or revitalization is widely described as the restoration of vascularity to a tissue or organ with the sole purpose of reestablishing the pulp-dentin complex, thereby contributing to repair and continued development of the necrotic immature permanent anterior teeth with respect to periapical healing, increase in root dimensions, and closure of the apical foramen [3].The current concept in regenerative endodontics primarily stems from the pioneering work done by Dr. B.W. Hermann wherein the meticulous application of calcium hydroxide for vital pulp therapy was described [4]. The judicious use of calcium hydroxide had gained acceptance worldwide and was also enlisted as the material of choice in apexification procedures before the advent of MTA [5]. However, the conventional endodontic approaches described above failed to offer any benefits as far as continued root development and thickening of root dentinal walls were concerned. As a result, a high incidence of root fracture could be elicited in such cases as reported by Andreasen et al. [6].

Reynolds et al. evaluated a revascularization protocol for rejuvenating the pulp-dentin complex in necrotic immature permanent teeth and reported a case of connective tissue formation in root canals following routine pulpectomy procedure [7]. Rule and Winter further strengthened the concept when pulpal bleeding was induced to revitalize a nonvital immature mandibular premolar [8]. This frank attempt tasted remarkable success when continued root formation and apical closure were detected. The concept of this “induced bleeding” technique has fast gained popularity over the years and catapulted regenerative endodontics into the limelight as an alternative to conventional root canal therapy when all else failed.

In recent years, various attempts have been made to regenerate the lost pulp which involved incorporation of popular platelet concentrates such as platelet-rich plasma (PRP) and platelet-rich fibrin (PRF). While the former suffers from few shortcomings pertaining to cost, handling, tedious centrifugation, and purification procedures, the latter scores over it in terms of simplicity and cost; the use of bovine thrombin and anticoagulants are also avoided [9]. Apart from the conventional platelet concentrates, CGF has been suggested as an ideal biomaterial as it releases growth factors that induces cell migration and proliferation and chemotactic activity on inflammatory cells and stimulates angiogenesis and tissue remodelling [10]. However, a comprehensive search of the literature revealed the absence of any publication regarding the use of CGF in pulp revitalization, thereby making it a novel platelet concentrate for application in regenerative endodontics. For describing the case reports, we have followed the checklist given in CARE guidelines (http://www.care-statement.org/) [11].

## 2. Case Presentation

### 2.1. Case Report 1

**Figure 1:**
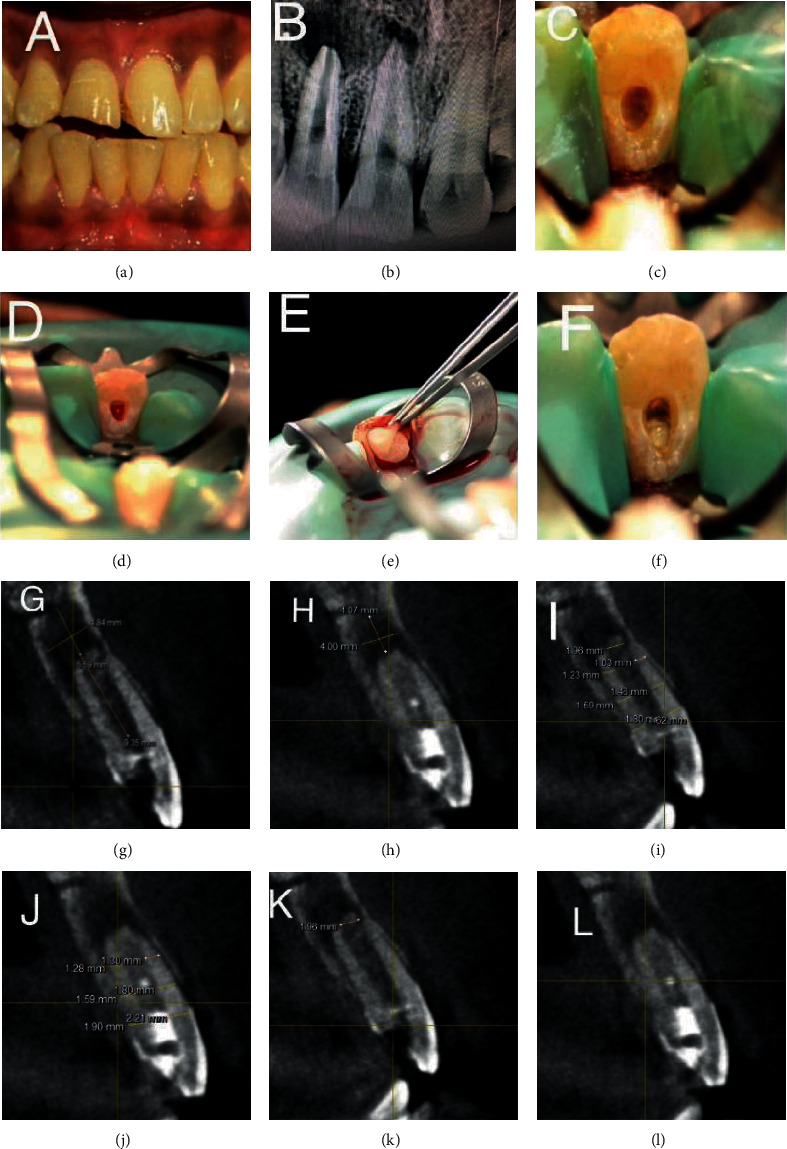
Preoperative clinical, revascularization procedure and radiographic images—IOPA and CBCT images (case report 1). (a) Pre-op clinical photograph showing fractured of 11 and 12. (b) Preoperative IOPA showing the periapical lesion involving the apices of 11 and 12 and immature roots of 12. (c) Access opening done in 12. (d) Bleeding induced in 12 for revascularization. (e) Placement of CGF in the root canal space. (f) MTA plug of 3 mm placed at the level of CEJ in 12. (g) Pre-op CBCT image showing the extent of lesion in sagittal slice. (h) Post-op CBCT image showing the extent of lesion in sagittal slice. (i) Pre-op CBCT showing the root dentin thickness measurement in sagittal slice. (j) Post-op CBCT showing the root dentin thickness measurement in sagittal slice. (k) Pre-op CBCT showing the apical diameter measurement in 12. (l) Post-op CBCT showing the apical diameter in 12.

A 23-year-old male patient reported to the Department of Conservative Dentistry and Endodontics with chief complaint of broken teeth and pain in the upper front tooth region. There was no contributing medical history. Past dental history revealed traumatic injury 14 years back associated with sharp pain on biting. Intraoral clinical examination revealed fracture of 11 and 12 (Figure 1(a)). Both the teeth were sensitive to percussion and palpation. Cold test was carried out using refrigerant spray (Coltene/Whaledent, Switzerland), and electric pulp testing (Gentle Pulse™ Pulp Vitality Tester, Parkell, USA) showed no response when compared with the control teeth. Periapical radiograph revealed loss of crown structure in 11, immature roots in 12, and radiolucency in the periapical area of 11 and 12 (Figure 1(b)). CBCT (Dentsply Sirona, Orthophos XG 3D) was taken at standardized settings (90 kV, 6 mA, 5∗5.5 cm, 160 *μ*m, 14 s). The lesion extent measured 4.84∗5.59∗9.35 mm (Figure 1(g)). Apical diameter measured 1.96 mm (Figure 1(k)). The root dentin thickness at coronal third was 1.62∗1.80 mm, middle third was 1.59∗1.46 mm, and apical third was 1.23∗1.03 mm (Figure 1(i)).

Based on these findings, the diagnosis made was pulpal necrosis with symptomatic apical periodontitis in 11 and 12. Revascularization protocol was applied for 12, and endodontic therapy was carried out in 11 with placement of intracanal medicament for three weeks (Figures 1(c)–1(f)). No adverse events were reported during the follow-up periods. Intraoral periapical radiograph and CBCT at 12-month follow-up revealed marked closure of the apex in 12 and decreased extent of periapical lesion in 11 and 12. The lesion extent measured 4.00∗4.07∗8.96 mm (Figure 1(h)). The root dentin thickness at coronal third was 1.90∗2.21 mm, middle third was 1.59∗1.80 mm, and apical third was 1.28∗1.30 mm (Figures 1(j)–1(l)).

**Figure 2:**
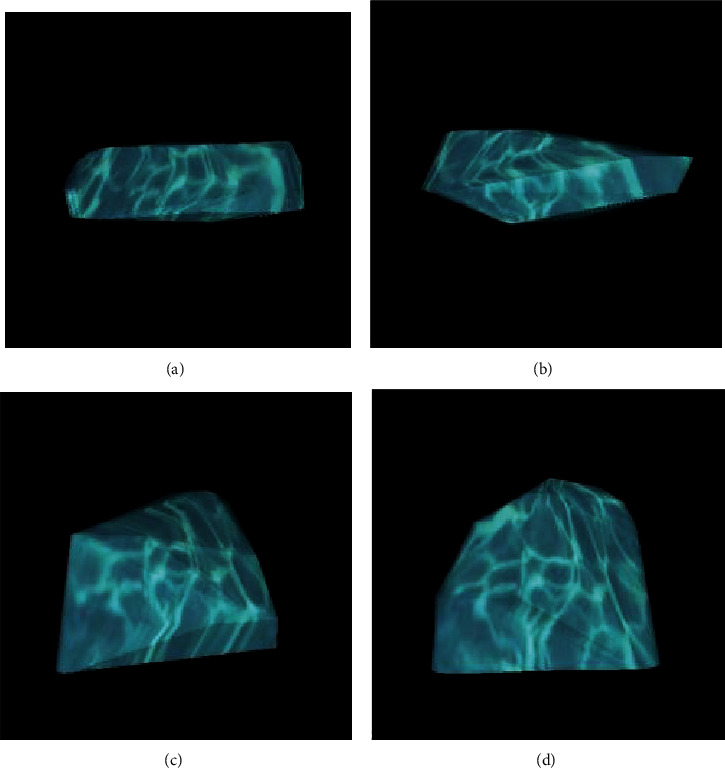
Preoperative and postoperative volume calculations to delineate the lesion from healthy bone. Case report 1: (a, b) preoperative and postoperative volume calculations, respectively. Case report 2: (c, d) preoperative and postoperative volume calculations, respectively.

Livewire segmentation using OSIRIX version 9.5 (PIXMEO, Geneva, Switzerland) was done to delineate the lesion from healthy bone. Preoperative and postoperative volume calculations were 0.1012cm^3^ and 0.0824 cm^3^, respectively (Figures 2(a) and 2(b)). The lesion reduction size was found to be 18.57%.

### 2.2. Case Report 2

**Figure 3:**
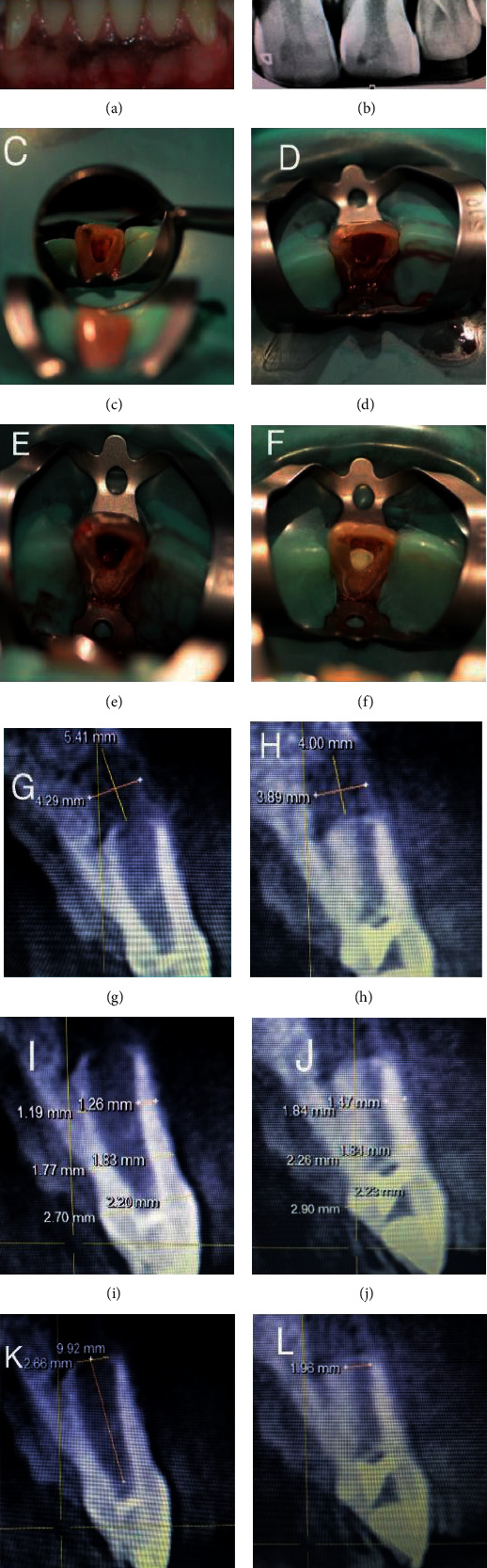
Preoperative clinical, revascularization procedure and radiographic images—IOPA and CBCT images (case report 2). (a) Pre-op clinical photograph showing discoloured and fractured 21 with sinus opening and pus discharge. (b) Preoperative IOPA showing periapical lesion involving the apices of 21 and open apex. (c) Access opening done in 21. (d) Bleeding induced in 21 for revascularization. (e) Placement of CGF in root canal space. (f) MTA plug of 3 mm placed at the level of CEJ in 21. (g) Pre-op CBCT image showing the extent of lesion in sagittal slice. (h) Post-op CBCT image showing the extent of lesion in sagittal slice. (i) Pre-op CBCT showing the root dentin thickness measurement in sagittal slice. (j) Post-op CBCT showing the root dentin thickness measurement in sagittal slice. (k) Pre-op CBCT showing the apical diameter measurement in 21. (l) Post-op CBCT showing the apical diameter in 21.

A 21-year-old male patient reported to the Department of Conservative Dentistry and Endodontics with chief complaint of discoloured and broken teeth in the upper front tooth region. Patient revealed a history of trauma 9 years back. Clinical examination revealed discoloured and fractured 21 with sinus opening and pus discharge (Figure 3(a)). On radiographic examination, incomplete root formation was evident in 21 with radiolucency in the periapical region (Figure 3(b)). The lesion extent in CBCT was measured to be 5.41∗4.29∗9.92 mm (Figure 3(g)). The root dentin thickness at coronal third was 2.70∗2.20 mm, middle third was 1.77∗1.83 mm, and apical third was 1.19∗1.26 mm (Figure 3(i)). Apical diameter measured 2.66 mm (Figure 3(k)). Based on these findings, a diagnosis of chronic apical abscess in 21 was made. Revascularization protocol was followed in 21 (Figures 3(c)–3(f)). No adverse events were reported by the patient during the follow-up periods. Intraoral periapical radiograph and CBCT at 12-month follow-up revealed increased root dentin thickness in 21. The lesion extent in CBCT was measured to be 4.00∗3.89∗8.98 mm (Figure 3(h)). The root dentin thickness at coronal third was 2.90∗2.23 mm, middle third was 2.26∗1.84 mm, and apical third was 1.84∗1.47 mm (Figure 3(j)). Apical diameter measured 1.96 mm (Figure 3(l)).

Preoperative and postoperative volume calculations were 0.0675cm^3^ and 0.0541 cm^3^, respectively (Figures 2(c) and 2(d)). The lesion reduction size was found to be 19.85%.

### 2.3. Common Treatment Protocol

Prior to the revascularization procedure, informed consent was obtained from the patient. On the first visit, teeth were isolated with a rubber dam, followed by access cavity preparation with endo access bur (Dentsply Maillefer, Switzerland) and working length determination using a #80 K-file (Mani, Inc., Japan) radiographically. The canals were irrigated using a 30-gauge side-vented needle with 20 ml of 1.5% sodium hypochlorite (VIP, Vensons, India) followed by saline rinse prior to the placement of calcium hydroxide (RC Cal, Prime dental products, India) as intracanal medicament for three weeks. For temporary sealing of the access cavity, zinc oxide eugenol was used. On the second visit, the patients were asymptomatic. 2% lignocaine (Lignox, India) without vasoconstrictor was administered. The medicament was flushed out of the root canal with copious amount of 0.9% saline (AcuLIFE, India). The root canal was then irrigated with 10 ml of 17% EDTA (Canalarge, Ammdent, India). Following this, the canals were dried with paper points, bleeding was induced using #80 K-file to a length of 2 mm beyond the working length, and the bleeding was made to fill the root canal. A cotton pellet was placed in the pulp chamber to allow clot formation. The protocol thus followed was in accordance with the guidelines proposed by the American Association of Endodontists (AAE).

### 2.4. Preparation of Concentrated Growth Factor

Two disposable 10 ml nonanticoagulant tubes and a matching centrifuge device (MEDIFUGE, Silfradent SRL, S. Sofia, Italy) were used. 10 ml of intravenous blood sample from the patient was obtained and placed in centrifuge tubes without anticoagulants; accelerated for 30 seconds; centrifuged at 2700 rpm for 2 min, 2400 rpm for 4 min, 2700 rpm for 4 min, and 3000 rpm for 3 min; and decelerated for 36 secs to stop. At the end of the automated preprogrammed cycle, the centrifuge tube encompassed a concoction of four different layers; the uppermost or the 1^st^ layer contained serum, the 2^nd^ layer was composed of a fibrin buffy coat, the 3^rd^ layer constituted the much needed growth factors, and lastly, the lowermost or the 4^th^ layer was occupied by the red blood cells. The fibrin gel containing the concentrated growth factor was separated from the red blood cells. The CGF obtained was packed into the canals to the level of cementoenamel junction. 3 mm MTA (MTA Angelus Brazil) plug was placed at the level of CEJ, and the coronal cavity was sealed using composite.

## 3. Discussion

The present case report most notably focused upon achieving revitalization at a flourishing rate, made possible by incorporating the novel autologous platelet concentrate (CGF), well within the guidelines proposed by the AAE. CGF was introduced by Sacco in 2006. As “concentrated” as it seems to be, CGF does stand out from its predecessors by the way it is concocted. This is ascribed to its unique centrifugation technique which depends upon varied time intervals and centrifugation speeds, finally churning out a much concentrated, thicker, and elongated fibrin matrix [12]. In a comparative study, it was claimed that CGF indeed had an upper hand over PRP and PRF in terms of cell proliferation and osteoblastic differentiation as well as a richer growth factor content [13].

In recent years, CGF has carved a niche for itself in terms of sinus ridge augmentation procedures, implant surgeries, and surgical endodontics as reported by Sohn et al., Qiao et al., Ying et al., and Sureshbabu et al., eliciting predictable and positive clinical impact in ridge augmentation and accelerated healing of intrabony defects, lateral cysts, and periapical pathologies, respectively [14–18]. In our recently published case report, we focused on the conspicuous coalescence of CGF and bone graft (sticky bone) to intercept a large periapical bony lesion, for which we garnered resounding success in stemming the repair and regenerative process [14]. Additionally, in our previously published clinical case presentation of two cases, we had implemented CGF as a scaffold in the surgical endodontic treatment procedures with great effect and were able to elicit successful healing outcomes [18]. Furthermore, Hong et al. reviewed the potential application of CGF and concluded that the autologous platelet concentrate could be a promising biomaterial in regenerative endodontics [19].

In case 1, routine IOPAR revealed a slight anatomical variation at the apex of 12 with periapical lesion which was followed by two parallax radiographs to confirm the same. Since periapical radiographs being a two-dimensional imaging modality, we could not ascertain as to whether the said tooth had a closed apical foramen and also could not assess the extent of the lesion in 11 and 12. In case 2, a single periapical radiograph confirmed the presence of blunderbuss canal with periapical lesion in 21. In both cases, the extent of lesion and apical diameter along with root dentin thickness had to be verified and measured with the aid of CBCT. According to AAE guidelines, CBCT scan with a small field of view is recommended in clinical research trials for diagnostic accuracy [20].

The use of anaesthetic solution without a vasoconstrictor was advocated in both our cases which was well in accordance with a study conducted by Petrino et al. [21]. It was reported that several attempts were made to induce bleeding into the canal space when anaesthetic solution containing a vasoconstrictor was employed. Jung et al. suggested that the induction of bleeding into the root canal provides stem cells that can induce dentin formation [22]. Similarly, in both our cases, bleeding was induced using a #80 K-file instrumented 2 mm beyond the working length. According to Wang et al., the outcome of revascularization procedure improved with the inclusion of blood clot in the root canal space which resulted in a process of repair rather than revitalization [23]. To that end, the autologous platelet concentrate has been effectively deployed in these cases as scaffold materials to compensate for the lack of formation of high-quality blood clots.

Rodella et al. demonstrated that growth factors TGF-ß1 and VEGF were found in abundance which according to the literature are highly essential for stimulating cell proliferation, matrix rehabilitation, and angiogenesis [24]. Based on an immunohistochemical analysis, the aforesaid study also confirmed the existence of circulating CD34+ cells in CGF and RBC layers which again is crucial for the revascularization process [25].

Disinfection is of prime importance in enhancing the predictability of regenerative endodontic procedures. According to Ruparel et al., Galler, and Segura-Egea et al., the use of antibiotics in regenerative endodontics should be avoided [26–28]. Althumairy et al. stated that disinfection with calcium hydroxide leads to a greater survival of SCAP cells as opposed to antibiotic pastes [29]. Irrigants are selected based on both their bacteriostatic or bactericidal properties and the ability to sustain the survival of stem cells. The chelating effect of 17% EDTA promoted the release of dentin-derived growth factors which in turn regulates the survival and differentiation of stem cells, whereas 2% chlorhexidine proved to be cytotoxic with no viable cells [30].

The biological mechanism of regeneration depends on the “self-renewal” [31] capacity of the stem cells of apical papilla (SCAP), capable of differentiating into odontoblast-like cells ultimately resulting in the formation of dentin-like tissue [32]. While treating nonvital immature permanent teeth, the preservation of SCAP promotes continuous formation of the root to its completion [33] Induced bleeding into the root canal space triggers higher concentration of mesenchymal stem cells (MSCs) from the apical papilla [34] that may not be sufficient for the formation of pulp-dentin complex; hence, autologous platelet concentrate as scaffold serves as a reservoir of growth factors, thus aiding in the regenerative process [35].

Histologic confirmation of dental pulp with an intact odontoblastic layer and restoration of a functional pulp is a pinnacle in regenerative treatment goals; therefore, further objective should be targeted at histological evaluation of viable pulp tissue [36]. The limitation of these case presentations is that it is not proved that true pulp-dentin complex regeneration has occurred for which histological examination is required. The effect of CGF in regenerative endodontics could be well established by randomized clinical trials and longer follow-up periods.

## 4. Conclusion

Although evidence concerning the implementation of CGF in revascularization procedures is scarce, however, based on available and limited data, it does deliver phenomenal success in diverse oral surgical applications (described earlier). In this case presentation, CGF was utilized with great effect as a fibrin-rich block, progressively releasing the much noteworthy growth factors to bring about a rapid and successful healing outcome. At one-year follow-up, CBCT assessment confirmed marked apical closure in case 1, increased root dentin thickness in case 2, and complete reduction in size of the periapical lesion in both cases. All things considered, CGF holds substantial promise for use in revascularization techniques to enhance the repair and regenerative process in a manner most beneficial in terms of all-round satisfactory healing of the immature permanent tooth.
